# New insights into the prognosis of intraocular malignancy: Interventions for association mechanisms between cancer and diabetes

**DOI:** 10.3389/fonc.2022.958170

**Published:** 2022-08-08

**Authors:** Lingwen Gu, Guofeng Ma, Cui Li, Jing Lin, Guiqiu Zhao

**Affiliations:** ^1^ Department of Ophthalmology, The Affiliated Hospital of Qingdao University, Qingdao, China; ^2^ Department of Urology, The Affiliated Hospital of Qingdao University, Qingdao, China

**Keywords:** intraocular malignancy, uveal melanoma, retinoblastoma, Warburg effect, insulin resistance, insulin-like growth factor-1 receptor

## Abstract

The intraocular malignancies, which mostly originate from the retina and uvea, exhibit a high incidence of blindness and even death. Uveal melanoma (UM) and retinoblastoma (RB) are the most common intraocular malignancies in adults and children, respectively. The high risks of distant metastases lead to an extremely poor prognosis. Nowadays, various epidemiological studies have demonstrated that diabetes is associated with the high incidence and mortality of cancers, such as liver cancer, pancreatic cancer, and bladder cancer. However, the mechanisms and interventions associated with diabetes and intraocular malignancies have not been reviewed. In this review, we have summarized the associated mechanisms between diabetes and intraocular malignancy. Diabetes mellitus is a chronic metabolic disease characterized by prolonged periods of hyperglycemia. Recent studies have reported that the abnormal glucose metabolism, insulin resistance, and the activation of the IGF/insulin-like growth factor-1 receptor (IGF-1R) signaling axis in diabetes contribute to the genesis, growth, proliferation, and metastases of intraocular malignancy. In addition, diabetic patients are more prone to suffer severe complications and poor prognosis after radiotherapy for intraocular malignancy. Based on the common pathogenesis shared by diabetes and intraocular malignancy, they may be related to interventions and treatments. Therefore, interventions targeting the abnormal glucose metabolism, insulin resistance, and IGF-1/IGF-1R signaling axis show therapeutic potentials to treat intraocular malignancy.

## Introduction

The intraocular malignancies primarily originate from the retina or uvea, which seriously impair vision and endanger life. Uveal melanoma (UM) is the most common intraocular malignancy in adults, with high risks of distant metastasis within 5 to 10 years. The liver is the most likely organ invaded for metastasis in UM. Retinoblastoma (RB) is the most common intraocular malignant tumor in children. The mortality of RB is up to 70% in low- and middle-income countries. However, the mechanisms of the genesis and metastases of intraocular malignancies are still under investigation. Current treatments (such as chemotherapy, radiotherapy, and enucleation) for intraocular malignancies patients with metastases are insufficient to improve the overall survival rate. Chemotherapy may cause myelosuppression or even secondary leukemia (1, 2). Serious complications associated with posterior pole abnormalities may occur after radiotherapy, leading to visual loss or even eyeball removal (3–5). It has been confirmed that patients with diabetes have a higher incidence of suffering severe complications after radiotherapy for intraocular malignancies (3). The average survival time of UM patients with metastases is less than half a year after diagnosis (6, 7). As for RB patients, the 5-year survival rate is as low as 30% in developing countries (8–10). Thus, it is of great significance to explore the factors associated with the genesis and metastases of intraocular malignancies to improve disease prognosis.

Diabetes mellitus is a metabolic disease characterized by long-term hyperglycemia accompanied by insulin resistance, hyperinsulinemia, and increased insulin-like growth factor (IGF)-1 levels (11–16). According to the statistics from International Diabetes Federation (IDF), there were 537 million people with diabetes mellitus and 6.7 million deaths from the disease in 2021, and the number of patients will rise to 783 million by 2045 (17). Epidemiological results have suggested that there is a strong association between diabetes and cancers (especially liver and pancreatic cancers) (18). A cohort study showed that the incidence of liver cancer in diabetic patients was 4.25 times higher than that in non-diabetic patients (19). Pang’s prospective study of half a million Chinese adults found that diabetes doubles the risk of pancreatic cancer (20). A recent report showed that cancer has become the leading cause of death in diabetic patients, surpassing cardiovascular diseases (21). For intraocular malignancies, a case–control study has demonstrated that the occurrence of unilateral RB is correlated with maternal diabetes (22). The retrospective clinical studies (23, 24) have shown that the tumor is more progressive in diabetic patients, compared to non-diabetic patients, implying that diabetes may lead to a poor prognosis of malignancies. The occurrence of diabetes shares various common risk factors with malignancies, including abnormal glucose metabolism, the activation of the IGF signaling axis, and insulin resistance (25, 26). Therefore, these factors may become the significant biological links between diabetes and malignancies, and the intervention of the above factors may bring new opportunities to the treatment of malignancies.

In this review, the common genesis mechanisms shared by diabetes and intraocular malignancies are summarized. According to recent studies, it can be noted that the IGF-1/insulin-like growth factor-1 receptor (IGF-1R) signaling axis is associated with the genesis and metastases of UM and RB (27, 28). In addition, abnormal glucose metabolism and insulin resistance contribute to the development of progressive intraocular malignancies (29–31). The above evidence shows that diabetes is associated with the genesis and poor prognosis of intraocular malignancies. Therefore, treatments targeting abnormal glucose metabolism, insulin resistance, and IGF-1/IGF-1R signaling axis may exert potential therapeutic effects to decrease the tumor burden and the metastasis risks of intraocular malignancies.

With the progression of diabetes, diabetic patients often suffer from ocular complications, such as diabetic retinopathy and macular edema, which can lead to decreased vision and metamorphopsia (32). These diabetic eye diseases also correlate to the prognosis of intraocular malignancies. For diabetic patients, it is essential to screen and monitor ocular complications during the early stage of diabetes. A retrospective case–control study showed that uveal melanoma patients detected during the screen of diabetic eye disease had a higher 5-year survival rate, which is associated with the earlier detection and treatment of tumors (33, 34). It is of great significance to diagnose and treat intraocular malignancies earlier to decrease the risks of metastases and mortality. In addition, diabetes may affect the therapeutic effect of radiotherapy for choroidal melanoma (35). Diabetic patients are more prone to suffer posterior pole abnormalities after radiation therapy, which is correlated to the retinal capillaries damage during diabetic retinopathy (35, 36). Thus, early screening and treatment of diabetic eye disease may improve the prognosis and survival rate of intraocular malignancies.

## Association mechanisms between diabetes and intraocular malignancy

Various studies have indicated that diabetes is involved in the increased incidence of cancer and mortality related to cancer (37–42). A variety of potential mechanisms contribute to the link between diabetes and cancer. Among them, abnormal glucose metabolism, insulin resistance, and increased IGF-1 levels are attracting increasing attention from researchers. Recently, studies have reported that the factors mentioned above also contribute to the genesis, growth, proliferation, and metastases of intraocular malignancies. In addition, patients with malignant tumors who undergo diabetes are at a higher risk of death after chemotherapy. For example, patients with diabetes who receive chemotherapy for pancreatic cancer have larger tumors and lower survival rates (hazard ratio 1.16, 95% confidence interval (CI) 1.08–1.25, p = 0.000) (43). Compared with cancer patients without diabetes, the risk of chemotherapy-induced neutropenia is 32% higher in cancer patients with diabetes (44). However, there are few studies investigating the effect of diabetes on intraocular tumor chemotherapy yet. More studies identify that the risks of severe complications after radiotherapy for intraocular malignancies are much higher in patients with diabetes.

### Abnormal glucose metabolism and intraocular malignancy

Glucose, as an important energy substance, plays a significant role in maintaining cellular metabolism in proliferating cells. Hyperglycemia can provide sufficient energy supply for the rapid proliferation, invasion, and metastasis of intraocular malignant tumor cells. The aerobic glycolysis is the main metabolism pathway of tumor cells, rather than the oxidative phosphorylation pathway, even under normoxic conditions. This characteristic metabolism approach of tumor cells is named the Warburg effect (45). Compared with the oxidative phosphorylation pathway, the production efficiency of ATP is lower in the aerobic pathway. Thus, to meet the huge demand for energy, a potent capacity of glucose uptake is required for the tumor cells (30). Diabetic patients are accompanied by hyperglycemia, providing sufficient energy for the growth of tumor cells. Hyperglycemia also leads to cancer progression by promoting cellular proliferation, metastasis activity, and inhibiting apoptosis of tumor cells (46). In addition, the intermediates produced by the Warburg effect provide metabolites for tumor cells, including amino acids, lipids, and other cellular energy substances, which can be used as precursors of anabolic pathways (47, 48).

**Figure 1:**
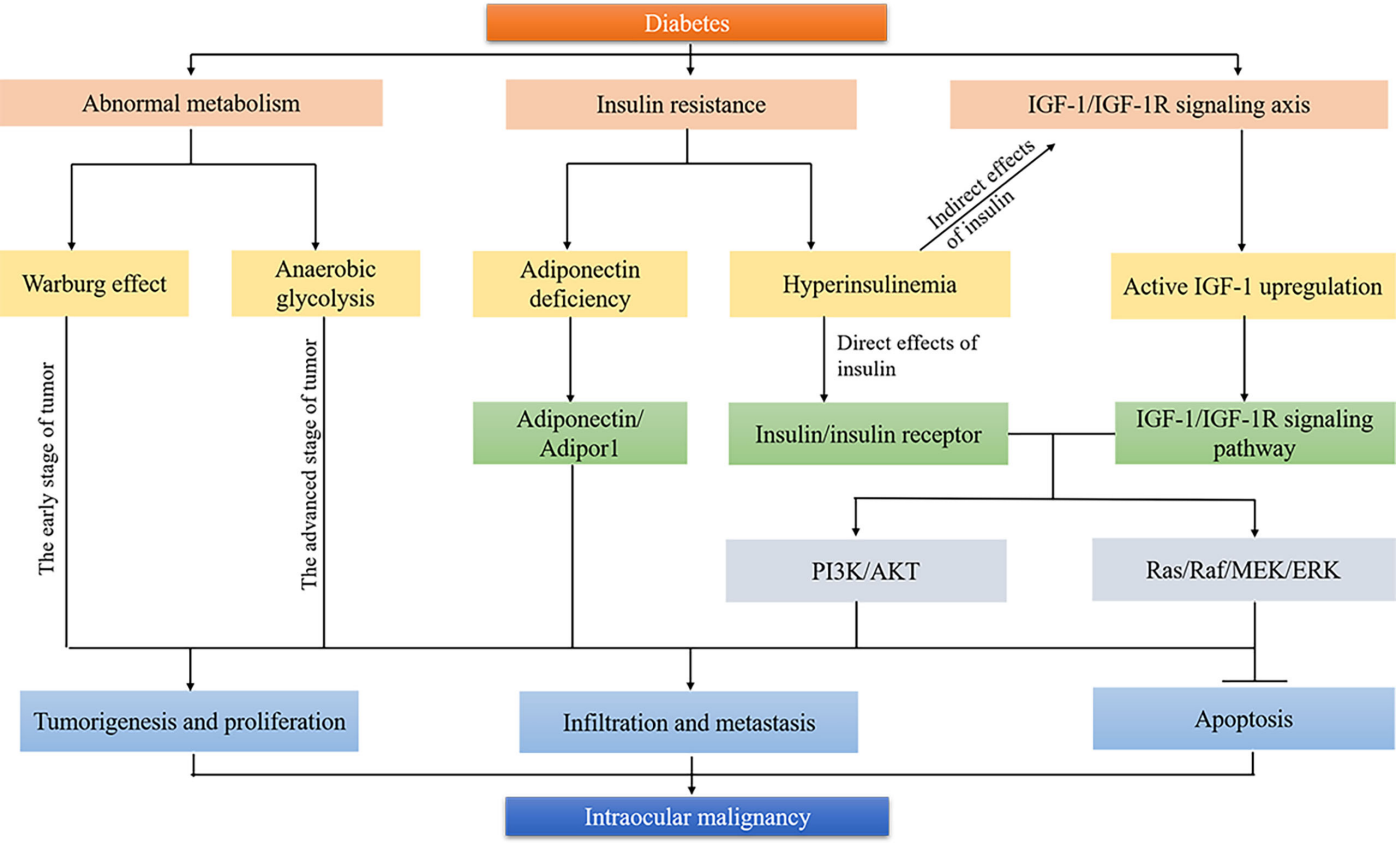
Biological mechanisms linking diabetes and intraocular malignancy. Tumor cells undergo metabolic reprogramming to meet their energy needs for rapid proliferation. Warburg effect and anaerobic glycolysis are involved in the formation and development of intraocular malignancies in the early stage and advanced stage, respectively. Insulin resistance improves carcinogenesis by developing an adiponectin-deficient environment, direct insulin/insulin receptor pathway, and indirect IGF-1/IGF-1R signaling pathway. Both IGF-1/IGF-1R axis and insulin/insulin receptor are involved in the occurrence and development of intraocular malignancy through PI3K/AKT and Ras/Raf/MEK/ERK signaling pathways. →, activation; ⊥, inhibition.

Metabolic reprogramming is essential for cancer progression, and tumor cells often undergo a series of metabolic reprogramming to meet their energy needs for rapid proliferation (49, 50). The UM cells display the Warburg effect, and the malignant degree of UM is associated with the activation of the aerobic glycolysis (30, 31). Metabolic enzymes related to aerobic glycolysis, including hexokinase 2 (HK2), glyceraldehyde-3-phosphate dehydrogenase (GAPDH), and lactate dehydrogenase (LDH), have been found to be linked to the growth of intraocular malignancies. The expression of these metabolic enzymes can be upregulated by activating oncogenic transcription factors, including hypoxia-inducible factor-1α (HIF-1a) and MYC (51, 52). As the rate-limiting enzyme for glycolysis, HK2 is overexpressed in UM patients. HK2 promotes tumor growth by maintaining a high glycolysis rate in fast-growing tumors, and the microRNA (miR)-216a-5p/HK2 axis plays a significant role in UM tumorigenesis (53). As an isoform of GAPDH, sperm-specific glyceraldehyde-3-phosphate dehydrogenase (GAPDHS) is also overexpressed in UM patients, which improves aerobic glycolysis, cellular growth, and proliferation of UM cells. Meanwhile, the downregulation of GPADHS limits aerobic glycolysis and tumor cell growth and proliferation (31). In the initial stages of tumorigenesis, a large amount of oxygen is required for tumor cells to survive and proliferate. However, in the later stages of tumor development, differences in oxygen metabolism result in a microenvironment of low oxygen and disorder microvasculature (54, 55). The development of RB is associated with the loss of anti-oncogene RB1, which affects the cell cycle, cellular proliferation, and apoptosis (56, 57). In addition to the RB1 gene, hundreds of genes are altered in RB. These genes can affect various signal transduction pathways (such as PI3K/AKT and p53), also contributing to the tumor microenvironment of hypoxia and high angiogenic activity, and abnormal glucose metabolism (58, 59). Due to the decreased proliferation activity of tumor cells in the hypoxic regions of RB, especially during the aggressive clinical course, they could hardly be killed by conventional treatments including chemotherapy and radiation (60). The tumor cells in the hypoxic region of intraocular malignancies at the advanced stage mainly rely on anaerobic glycolysis for energy supply. **Figure 1** demonstrates that the Warburg effect and anaerobic glycolysis of tumor cells were involved in the formation and development of intraocular malignancies in the early stage and advanced stage, respectively.

In addition to the key metabolic enzymes related to aerobic glycolysis, glucose transporter (GLUT) proteins are significant for the genesis and progression of UM, although diabetic patients with hyperglycemia can provide sufficient energy for cellular growth and metastasis of UM cells. The hydrophilic glucose cannot passively penetrate the hydrophobic plasma membrane into the cell. Thus, high expression of GLUT proteins is required for tumor cells to meet the high level of glucose translocate and uptake. A previous study has identified the overexpression of GLUT1 protein in UM cells under hypoxia conditions (61). In addition, the downregulation of hypoxia-response genes, including cyclic Amp-Response-Element binding protein (CREB) and HIF-1, has been demonstrated to reduce GLUT1 protein expression in a mouse model of UM and slow tumor progression (62).

### Insulin resistance and intraocular malignancy

Insulin resistance and compensatory hyperinsulinemia are present for a long period of time before the development of hyperglycemia in type 2 diabetes patients (63–65). Insulin resistance could induce hyperinsulinemia due to the excess production of insulin by pancreatic β-cells. Pancreatic β-cells finally decompensate, and hyperglycemia occurs (13). Insulin resistance and hyperinsulinemia have been proposed as crucial factors related to the high incidence and mortality of cancers (such as breast, pancreatic and colorectal cancers) in patients with diabetes (14, 66, 67). Insulin resistance can improve carcinogenesis by direct insulin/insulin receptor pathway and indirect IGF signaling pathway (**Figure 1**). Insulin resistance promotes the overproduction of IGF-1 and IGF-2 due to the limitation of IGFBP. Both insulin/insulin receptor and IGF signaling pathways lead to the activation of insulin response substrate-1 (IRS-1) and the receptor tyrosine kinase pathway, including Ras/Raf/MAPK kinase/extracellular signal-regulated kinase (ERK) cascade, which is essential for carcinogenesis by promoting the proliferation and protein synthesis and inhibiting apoptosis of tumor cells (68, 69). Recently, insulin resistance has gained attractive attention in the growth and progression of intraocular malignancies.

Evidence has shown that insulin resistance is associated with the genesis and the aggressive clinical course of intraocular malignancies (29). UM, the most common intraocular malignancy in adults, can be transformed from choroidal nevi and result in systemic metastasis eventually (70). A previous study detected the insulin levels and insulin resistance scores in patients with UM and choroidal nevi and patients in the control group. Results showed that compared with the control group, both insulin levels and insulin resistance scores were higher in patients with UM or choroidal nevi. The insulin levels and insulin resistance scores were the highest in patients with UM (29). Insulin resistance promotes the progression of UM mainly by enhanced expression of IGF-1, which is closely associated with scleral infiltration and systemic metastasis in UM (71–74). Insulin resistance also aggravates the course of UM by developing an adiponectin-deficient environment. Adiponectin is a hormone that enhances insulin sensitivity and exerts antitumor function by binding its receptor on tumor cells (75, 76). Aysegül Tura et al. indicated the low expression of adiponectin and Adipor1 (the receptor of adiponectin) in UM patients (77). The lack of adiponectin and Adipor1 can improve the metastasis ability of UM cells and terminate tumor cell dormancy (**Figure 1**). In addition, insulin resistance could further progress to hyperinsulinemia, contributing to tumorigenesis by activation of mitogenic signaling and limitation of apoptosis. Thus, avoiding insulin resistance may limit the growth and metastases of intraocular malignancies.

### IGF-1/IGF-1R signaling axis and intraocular malignancy

The main pathophysiological change of type 2 diabetes is insulin resistance, which leads to hyperinsulinemia. In addition to the direct effect of insulin, IGF-1/IGF-1R signaling axis activation plays a crucial role in the occurrence and development of intraocular malignancies. IGF-1 is a critical regulator associated with energy metabolism and cell proliferation with high homology with insulin precursors (29). By binding to IGF-1R, IGF-1 activates mitogen-activated protein kinase (MAPK) and phosphatidylinositol 3 kinase (PI3K) signaling pathways, which correlate with neogenesis (78). In body fluids, most of the IGF-1 exists without activity due to the combination with IGF binding protein (IGFBP). IGFBP has a great affinity for IGF-1 and can inhibit the activity of IGF-1 by limiting the combination of IGF-1 and IGF-1R. Due to the high level of circulating insulin in patients with type 2 diabetes, the production of IGFBP-1 is restricted. Decreased IGFBP-1 leads to the upregulation of free IGF-1 and the activation of an IGF-1/IGF-1R signaling pathway (79). The activation of the IGF-1/IGF-1R signaling pathway may activate the MAPK and PI3K signaling pathways that are closely related to tumor genesis, resulting in the occurrence of tumor. In fact, high expression of IGF-1 and IGF-1R has been indicated to contribute to the increased risk of a variety of cancers, including colorectal, gastric, breast, ovarian, bladder, kidney, and prostate cancers (80, 81). Recent studies have found that the IGF-1/IGF-1R signaling axis was also associated with the genesis and metastasis of UM and RB (**Figure 1**).

Obaa et al. observed that the IGF-1 level was higher in metastatic UM patients, compared with normal individuals, indicating that IGF-1 may regulate the invasion and metastasis of UM (82, 83). Compared with the UM cell lines with low expression of IGF-1R, high expression of IGF-1R is strongly associated with the prognosis of UM (84, 85). In addition, Economou et al. found that more patients died due to metastatic UM in the group with higher IGF-1R expression than in the group with lower IGF-1R expression (31). The liver is usually the most likely site for the metastases of UM. This could be explained by the high expression of IGF-1 in the liver since IGF-1 is mainly synthesized and secreted by liver cells. Thus, patients with high expression of IGF-1R are prone to suffer tumor metastasis, and IGF-1R levels could be used to predict the prognosis and survival of UM patients. Except for UM, IGF-1R also plays an oncogene role in RB, which can regulate the growth, migration, and invasion of RB cells. IGF-1R has been proposed to be a potential target of miR−98, and the dysregulation of miR−98 is involved in the occurrence and development of RB. A previous study has identified that miR−98 could restrict the growth and metastasis of RB tumor cells by regulating IGF-1R/Ras/Raf signaling pathway. However, the anti-RB functions of miR−98 were reversed after the restoration of IGF-1R (28). These results underlie that IGF-1R exerts a significant role in the genesis and metastasis of UM and RB.

### Diabetes affects the prognosis of intraocular malignant patients after radiotherapy

Moreover, diabetes is associated with the poor prognosis of intraocular malignancies patients after radiotherapy. Though radiotherapy could destruct tumor tissues, it can also cause serious complications, including radiation retinopathy (RR) and radiation optic neuropathy (RON). These complications are the most common causes of long-term vision loss and secondary eyeball removal for patients with intraocular malignancies after radiotherapy (86).

A randomized, multicenter clinical trial has indicated that diabetes is one of the major prognostic factors for posterior pole abnormalities after radiotherapy (3). Compared to non-diabetic patients, patients with diabetes are at higher risk of RR and RON. This could be explained by the vasculopathy caused by diabetes. Both radiation and diabetes damage the main components of retinal capillaries, such as endothelial cells and pericytes. Once the retinal capillaries lose cellular support, vascular occlusion, leakage, and hemorrhage may occur (36). Due to the combined action of diabetes and radiotherapy, patients with diabetes showed a more serious radiation vasculopathy than non-diabetic patients. Thus, in order to reduce the incidence of RR and RON in patients with intraocular malignancies after radiotherapy, attention should be paid to keeping the blood glucose within the normal range.

## Effects of interventions associated with diabetes on intraocular malignancy treatment

Since the occurrence and development of diabetes and intraocular malignancy share common pathogenic factors, they could be related in terms of the interventions and treatments. Therefore, interventions targeting the pathogenic factors of diabetes may bring benefits to the prognosis of intraocular malignancies.

### Treatments targeting the abnormal glucose metabolism limit energy supply and growth of intraocular tumor cells

**Figure 2:**
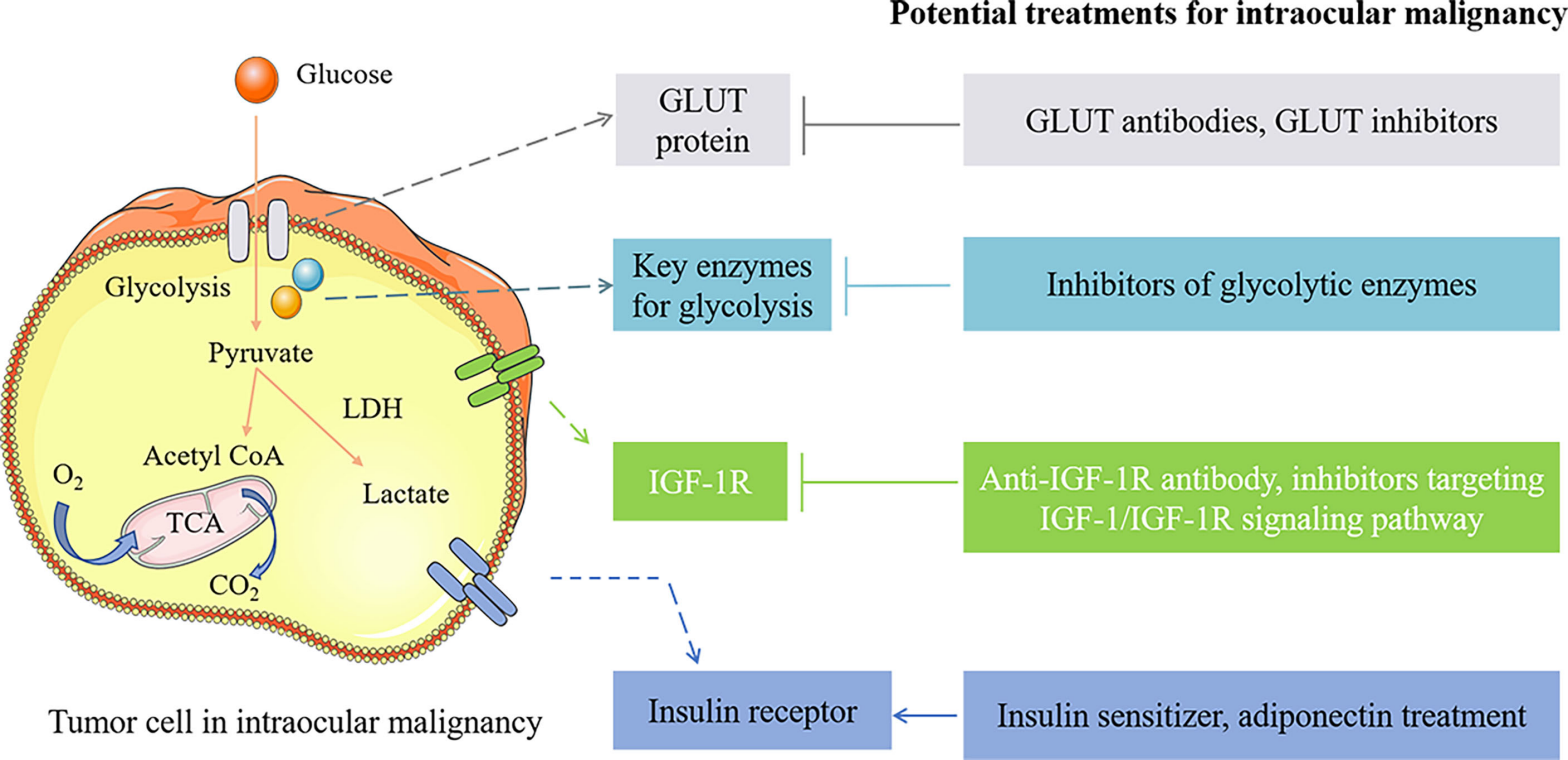
The potential treatments for intraocular malignancy by intervening the pathogenic factors of diabetes. GLUT1 inhibitors as well as GLUT1 antibodies inhibit tumor cell growth by limiting glucose translocated into the cell. Treatments targeting glycolytic enzymes inhibit glucose metabolism and tumorigenesis, including miR-216a-5p (the HK2 inhibitor), SOX-10 knockdown (GAPDHS suppression), and 2-DG and 2-FG (glycolytic inhibitors). Anti-IGF-1R antibody, IGF-1R inhibitor (such as pristimerin), PI3K/AKT inhibitors, FoxO3a overexpression, and miR-98 upregulation exert antitumor function by limiting IGF-1R and IGF-1/IGF-1R signaling pathway. Insulin sensitizer (such as metformin) and adiponectin treatment are considered potential approaches for treating intraocular malignancies by improving insulin resistance. →, activation; ⊥, inhibition.

The abnormal glucose metabolism of intraocular malignancy is characterized by the Warburg effect. Even under aerobic conditions, aerobic glycolysis is the main glucose metabolism pathway for tumor cells, and a large amount of glucose is required for energy supply through the Warburg effect. Hyperglycemia in diabetes patients provides sufficient energy substance for tumor cells, promoting tumor growth and proliferation. Various proteins and metabolic enzymes participate in the Warburg effect of cancers, including GLUT1 and numerous glycolytic enzymes, such as HK2 and GAPDH. Thus, novel treatments targeting the metabolic enzymes and proteins, inhibiting the process of aerobic glycolysis, and promoting the transformation from glycolysis to mitochondrial respiration are of great significance for treating intraocular malignancies (**Figure 2**).

The glycolytic enzymes are noted as the targets to treat intraocular malignancies by limiting glucose uptake, ATP production, and tumor genesis. HK2 is a key enzyme in the glycolysis process, catalyzing the initial and rate-limiting step of glycolysis (87–89). HK2 has been indicated to be overexpressed in cancers and related to the aggressive clinical course (**Figure 2**). By targeting the 3′-UTR structure of UM tumor cells, miR-216a-5p can inhibit HK2 excess expression. *In vitro* and *in vivo* experiments have shown that the miR-216a-5p supplement suppresses the growth and proliferation of UM by inhibiting the HK2-mediated Warburg effect (53). The miR-216a-5p/HK2 axis is closely associated with glucose uptake, lactic acid production, and ATP production of tumor cells. Therefore, approaches targeting HK2 overexpression are expected to be a promising strategy for treating UM. As an isoform of GAPDH, GAPDHS is associated with tumorigenesis by involving the Warburg effect of UM tumor cells. GAPDHS can be activated by Sry (sex determining region Y)-related HMG box 10 (SOX-10), which is significant for the survival and migration of melanocytes (90). Researchers have indicated that SOX-10 knockdown efficiently limited intraocular malignancy growth and promoted the malignant phenotype of tumor cells by inhibiting the GAPDHS-related Warburg effect, providing a novel target for treating UM (31). In the later stages of intraocular malignancies, due to the microenvironment alteration, hypoxic regions are formed where the tumor cells rely on anaerobic glycolysis for energy supply. Thus, treatments targeting glycolysis in the hypoxic region may exert antitumor function in the later stages of tumor growth. Previous studies have demonstrated that glycolytic inhibitors exhibit efficient therapeutic potentials to treat the hypoxic regions in RB (91, 92). For instance, the glycolytic inhibitor 2-deoxy-d-glucose (2-DG) treatment could influence the gene expression associated with metabolism, hypoxia, and angiogenesis and reduce RB tumor burden (91, 93). 2-DG can halt the glycolysis of hypoxic tumor cells by inhibiting HK enzyme activity (91). As a more efficient glycolytic inhibitor, 2-deoxy-2-fluoro-d-glucose (2-FG) treatment displayed a stronger effect of reducing tumor burden, providing a beneficial approach to treating RB (60). Treatments targeting glycolysis also helped to deal with the drug resistance of intraocular tumors related to hypoxic regions (94).

In addition, GLUT1-mediated glucose transport is crucial for the energy metabolism of tumor cells. The inhibitors of GLUT1, as well as GLUT1 antibodies, could inhibit the growth of tumor cells and display synergism effects with chemotherapy drugs (95–97). Tjorge et al. noted that GLUT1 expression was elevated in UM cells with monosomy-3, which could increase the metastatic possibility by upregulating glucose uptake of UM cells (98). These results indicate that GLUT may become the potential target to limit tumor growth and improve the prognosis of UM (**Figure 2**).

Tumor cells of ocular malignancies undergo metabolic reprogramming to meet the demand for energy. Studies have indicated that abnormal glucose metabolism including the Warburg effect and anaerobic glycolysis at different stages are associated with the survival and development of UM and RB. Thus, treatments targeting abnormal metabolism can greatly inhibit the growth of tumor cells and significantly improve the prognosis of intraocular malignancies.

### Treatments improving insulin resistance block cell cycle and induce apoptosis in intraocular tumor cells

Patients with type 2 diabetes are accompanied by insulin resistance. Insulin resistance is involved in the formation and aggressive course of intraocular malignancies by improving IGF-1 expression, decreasing adiponectin levels, and limiting apoptosis of tumor cells. Thus, it is necessary to pay attention to whether patients with ocular tumors are accompanied by insulin resistance. Treatments improving insulin sensitivity may exert therapeutic effects on patients with intraocular malignancies (**Figure 2**).

Metformin is a kind of insulin sensitizer widely used to treat type 2 diabetes. Libby et al. conducted a cohort study to detect the effects of metformin on the aggressive level of cancers. Their results indicated that the risk of cancer-related mortality was much lower in diabetic patients with metformin treatment than in diabetic patients with sulfonylureas or exogenous insulin treatments. Previous studies showed that metformin therapy decreased the risk of colorectal adenoma (OR = 0.73, CI 0.58, 0.90), liver cancer (OR = 0.52, CI 0.40, 0.68), and lung cancer (risk ratio = 0.89, CI 0.83, 0.96) (99–101). The above results suggest that metformin may exert a therapeutic effect on intraocular tumors. This protective role of metformin in cancers is thought to be related to its secondary effects of counteracting insulin resistance and improving insulin sensitivity and the expression of IGF1 and IGFBP3 (102, 103). Researchers have demonstrated that metformin could inhibit the proliferation of RB cells by blocking the cell cycle by regulating AMP−activated protein kinase (AMPK), mTOR signaling pathway, and autophagy process. Metformin has also been indicated to exhibit anti-proliferative and anti-migrative roles in ocular melanoma cells. *In vitro* and *in vivo* experiments both demonstrated that the tumor-specific inhibition of metformin is associated with autophagic flux limitation *via* silencing optineurin (OPTN) (104). OPTN is an oncogene in ocular malignancies, which can lead to tumorigenesis. However, overexpression of OPTN can impair the antitumor function of metformin by reducing metformin-induced autophagic inhibition. These studies provide evidence that metformin may be served as a novel treatment for intraocular malignancies.

Adiponectin is a hormone that could elevate the sensitivity of insulin. A great number of evidence has shown that adiponectin presents a protective role in cancers due to its antagonist effect on insulin resistance (67, 105). In patients with insulin resistance or type 2 diabetes, the adiponectin expression is decreased. A recent study has notified that adiponectin may exert protective effects to delay the metastasis of intraocular malignancies. Compared with the control group, serum adiponectin level is lower in UM patients or choroidal nevus patients. Moreover, the serum adiponectin level is reduced significantly in UM patients with systemic metastases, compared with non-metastases UM patients (67). These results indicate that the reduced expression of adiponectin may lead to insulin resistance and contribute to a more aggressive state of the disease by exerting proangiogenic and antiapoptotic effects (29). Adiponectin treatment could induce a quiescent phenotype of tumor cells in ocular tumors and effectively inhibit tumor growth by regulating insulin resistance and decreasing the proliferation marker and cell cycle of tumor cells (67). When adiponectin is severely deficient in UM cells, metformin is thought to inhibit the proliferation of UM cells by upregulating adiponectin expression. Thus, the upregulation of adiponectin and the counteraction of insulin resistance are considered potential approaches to delay UM metastasis (**Figure 2**).

In addition to treatments improving insulin sensitivity, lifestyle needs to be altered to counteract insulin resistance and adiponectin deficiency to improve the prognosis of intraocular malignancies. Studies have shown that increased intake of fiber, fish, vegetables, and fruits; adherence to aerobic exercise; and avoidance of smoking help to increase circulating adiponectin (106–108), though treatments targeting insulin resistance have not been used for UM and RB patients yet. More in-depth studies are required to indicate the effects of insulin resistance on the pathophysiology of intraocular malignancies.

### Treatments targeting IGF-1/IGF-1R signaling axis inhibit intraocular tumor cell proliferation and invasion

Due to insulin resistance and hyperinsulinemia, the IGF-1/IGF-1R signaling axis is activated in patients with diabetes. IGF-1/IGF-1R plays a significant role in the occurrence and development of intraocular malignancies. Thus, the IGF-1/IGF-1R axis may be involved in the tumorigenesis and metastasis of intraocular malignancies in diabetic patients. Targeting the IGF-1/IGF-1R signaling axis may exert therapeutic effects on intraocular malignancies (**Figure 2**).

IGF-1 is a kind of growth factor that can regulate the cell cycle and initiate the proliferation, migration, and invasion of intraocular malignancies by binding to its receptor. IGF-1R is highly expressed in UM and RB and is closely related to liver metastasis. Increasing evidence has indicated the IGF-1/IGF-1R axis as a target for treating intraocular malignancies. **Table 1** summarizes the drugs/inhibitors or molecules targeting the IGF-1/IGF-1R signaling axis, which can affect the genesis or prognosis of intraocular malignancies. The anti-IGF-1R antibody inhibits the proliferation and metastasis of UM cells induced by IGF-1 (109). The anti-IGF-1R antibody was also applied in a phase II study in patients with UM (110). The downstream IGF-1 and IGF-1R/PI3K/Akt signaling pathway are activated in UM, contributing to the tumorigenesis process (114, 115). Since the prominent role of IGF-1R/PI3K/Akt signaling pathway in tumorigenesis, PI3K and Akt inhibitors were used to treat UM. Results showed that PI3K and Akt inhibitors can effectively block the effects of IGF-1 including UM cell proliferation and invasion (111, 116, 117). FoxO3a, a kind of transcription factor, can be phosphorylated by IGF-1 *via* PI3K/Akt pathway in UM. Previous results have indicated that the inhibition of FoxO3a participated in the IGF-1-induced tumor growth and migration process. Thus, FoxO3a overexpression is beneficial to decrease the basal invasion of UM, providing a novel target for treating and preventing UM (76). Xie et al. have tested the antitumor function of pristimerin *via* IGF-1 and IGF-1R-mediated signaling pathways. Pristimerin has been indicated as a novel promising IFG-1R inhibitor. Results indicated that pristimerin suppressed proliferation and invasion of UM cells by downregulating IGF-1R and the downstream IGF-1R/Akt/mammalian target of rapamycin (mTOR) and ERK1/2 pathways (112). A previous study demonstrated that picropodophyllin (another inhibitor of IGF-1R) could cause UM tumor regression and reduce the risk of tumor metastasis (113). In addition to UM, IGF-1R has been proposed as a promising therapeutic target for RB. MiR-98, a kind of microRNA, can inhibit the proliferation, migration, and invasion of tumor cells (118). Previous studies have shown that miR-98 is involved in the development of RB by targeting IGF-1R and the downstream Ras/Raf/MEK/ERK signaling pathway. During the tumorigenesis of RB, the expression of miR-98 is decreased in RB tissues and tumor cells. However, upregulation of miR-98 effectively limits tumor growth and invasion and improves the prognosis of RB by targeting IGF-1R (28). These findings suggested that miR-98 may exert therapeutic function for patients with RB *via* inhibiting IGF-1R. The combination of IGF-1R inhibitor with the inhibitor targeting downstream signaling pathway also helps to decrease the risks of metastases, providing a potential approach to improve the outcome of intraocular malignancies.

**Table 1:** Drugs/inhibitors or molecules targeting the IGF-1/IGF-1R signaling axis which affect diabetes and intraocular malignancy.

Drug/inhibitor or molecule name	Shared pathological molecules/proteins	Signaling pathway	Effect on intraocular malignancy	Reference
IMC-A12	IGF-1R (anti-IGF-1R antibody)	IGF-1/IGF-1R signaling pathway	IMC-A12 suppressed the proliferation and metastasis of UM cells	(109)
			IMC-A12 was applied in patients with metastatic UM for phase II study	(110)
LY 294002	PI3K (PI3K inhibitor)	IGF-1R/PI3K/Akt signaling pathway	LY 294002 inhibited G1 CDKs in choroidal melanoma cells	(111)
Pristimerin	IGF-1R (IGF-1R inhibitor)	IGF-1R/Akt/mTOR and ERK1/2 pathways	Pristimerin limited proliferation and invasion of UM cells	(112)
Picropodophyllin	IGF-1R (IGF-1R inhibitor)	IGF-1R, Akt, ERK signaling pathways	Picropodophyllin blocked cellular growth of UM cells and delayed tumor formation in xenografted mice	(113)
FoxO3a	IGF-1	IGF-1/IGF-1R/PI3K/Akt signaling pathway	FoxO3a overexpression helped to alleviate the basal invasion of UM	(76)
MiR-98	IGF-1R	IGF-1R and Ras/Raf/MEK/ERK signaling pathways	Upregulation of miR-98 significantly suppressed tumor growth and improved the prognosis of RB	(28)

These results demonstrate that the IGF-1/IGF-1R signaling axis is involved in the formation and metastasis of intraocular malignancies. Thus, the approaches targeting the IGF-1/IGF-1R signaling pathway and the downstream effectors of the IGF-1/IGF-1R axis may exert potential therapeutic effects for intraocular malignancies.

## Discussion

Intraocular malignancies are a kind of sight- and life-threatening aggressive diseases. Among them, UM and RB are the most common intraocular malignancies in adults and children, respectively. Although local treatments such as chemotherapy and radiotherapy are available for treating intraocular tumors, the mortality still remains extremely high due to the invasion and metastasis of these diseases. Recent studies have indicated that the genesis and prognosis of intraocular malignancies may be related to diabetes. Thus, focusing on the association mechanisms between diabetes and tumors and intervening in related targets may bring opportunities to treat intraocular malignancies.

In contrast to type 2 diabetes, patients with type 1 diabetes mainly present with hyperglycemia and insulin deficiency, accounting for only 5%–10% of the total number of patients with diabetes (32). Since most patients with type 1 diabetes are younger than type 2 diabetes patients, longer-term follow-up studies are required (13). There is less literature supporting the relationship between type 1 diabetes and intraocular malignancies. Thus, our review is mainly based on type 2 diabetes. Insulin resistance, reduced adiponectin, and other mechanisms of intraocular tumor genesis are associated with type 2 diabetes. In addition, various studies have identified that the type 2 diabetes drug metformin helps to limit the growth and invasion of intraocular tumor cells.

In our paper, we propose a framework for considering the impact of diabetes on the occurrence, development, and prognosis of intraocular malignancies, based on the biological association between them. We discuss the biological causes of abnormal glucose metabolism, activation of IGF-1/IGF-1R signaling axis and insulin resistance in diabetic patients, and how they promote the occurrence and metastasis of UM and RB, suggesting that diabetes may have an impact on the development and prognosis of intraocular malignancies. In fact, hyperglycemia in diabetic patients provides a sufficient energy source for tumorigenesis. Intraocular tumor cells undergo metabolic reprogramming to meet their energy needs for rapid proliferation, such as the Warburg effect. Thus, novel approaches targeting the metabolic enzymes and proteins, and inhibiting the Warburg effect are of great significance for treating intraocular malignancies. IGF-1/IGF-1R axis contributes to the proliferation and invasion of tumor cells. The inhibitors targeting the IGF-1/IGF-1R axis and the downstream signaling pathway help to decrease the risks of metastases of intraocular malignancies. Insulin resistance is also involved in the formation and aggressive course of intraocular malignancies by enhancing IGF-1 expression, decreasing adiponectin levels, and limiting apoptosis of tumor cells. Treatments counteracting insulin resistance and adiponectin application may improve the prognosis of intraocular malignancies. Moreover, diabetes is associated with the poor prognosis of intraocular malignancies patients after radiotherapy.

This review indicates the biological link between diabetes and intraocular malignancies. Prevention of diabetes and treatments targeting abnormal glucose metabolism, IGF-1/IGF-1R signaling axis, and insulin resistance may provide novel opportunities to improve prognosis and delay the lethal metastases of intraocular malignancies.

## Author contributions

LG drafted the original manuscript. GZ conceived and supervised the study. GM conducted the literature search. CL conducted the picture processing. JL revised the manuscript. All authors contributed to the article and approved the submitted version.

## Funding

This work was financially supported by the National Natural Science Foundation of China (Nos. 81870632 and 81800800) and the Taishan Scholars Program (Nos. ts201511108 and tsqn202103188).

## Conflict of interest

The authors declare that the research was conducted in the absence of any commercial or financial relationships that could be construed as a potential conflict of interest.

## Publisher’s note

All claims expressed in this article are solely those of the authors and do not necessarily represent those of their affiliated organizations, or those of the publisher, the editors and the reviewers. Any product that may be evaluated in this article, or claim that may be made by its manufacturer, is not guaranteed or endorsed by the publisher.

## Abbreviations

UM, uveal melanoma; RB, retinoblastoma; IGF, insulin-like growth factor; IGF-1R, insulin-like growth factor-1 receptor; IDF, International Diabetes Federation; HK2, hexokinase 2; GAPDH, glyceraldehyde-3-phosphate dehydrogenase; LDH, lactate dehydrogenase; TCA, tricarboxylic acid cycle; HIF-1a, hypoxia-inducible factor-1α; miR, micro RNA; GAPDHS, sperm-specific glyceraldehyde-3-phosphate dehydrogenase; GLUT, glucose transporter; CREB, cyclic Amp-Response-Element binding protein; ERK, extracellular signal-regulated kinase; MAPK, mitogen-activated protein kinase; PI3K, phosphatidylinositol 3 kinase; IGFBP, IGF binding protein; RR, radiation retinopathy; RON, radiation optic neuropathy; SOX-10, Sry (sex determining region Y)-related HMG box 10; 2-DG, 2-deoxy-d-glucose; 2-FG, 2-deoxy-2-fluoro-d-glucose; AMPK, AMP−activated protein kinase; OPTN, optineurin.
